# Antimicrobial and mechanical properties of functionalized textile by nanoarchitectured photoinduced Ag@polymer coating

**DOI:** 10.3762/bjnano.14.11

**Published:** 2023-01-12

**Authors:** Jessica Plé, Marine Dabert, Helene Lecoq, Sophie Hellé, Lydie Ploux, Lavinia Balan

**Affiliations:** 1 Université d’Orléans, Conditions Extrêmes Matériaux Haute Température et Irradiation CNRS UPR 3079, F-45000, Orléans, France; 2 Biomaterials Bioengineering INSERM/Université de Strasbourg U1121, Centre de Recherche en Biomédecine de Strasbourg, F-67000 Strasbourg, France; 3 Université de Strasbourg, Faculté Dentaire, F-67000 Strasbourg, France; 4 CNRS, F-67000 Strasbourg, France

**Keywords:** antimicrobial properties, *C. albicans* fungus, *E. coli* bacteria, photoinduced functionalized textile, silver/polymer nanomaterials

## Abstract

The control of microbial proliferation is a constant battle, especially in the medical field where surfaces, equipment, and textiles need to be cleaned on a daily basis. Silver nanoparticles (AgNPs) possess well-documented antimicrobial properties and by combining them with a physical matrix, they can be applied to various surfaces to limit microbial contamination. With this in mind, a rapid and easy way to implement a photoinduced approach was investigated for textile functionalization with a silver@polymer self-assembled nanocomposite. By exposing the photosensitive formulation containing a silver precursor, a photoinitiator, and acrylic monomers to a UV source, highly reflective metallic coatings were obtained directly on the textile support. After assessing their optical and mechanical properties, the antimicrobial properties of the functionalized textiles were tested against *Escherichia coli (E. coli) and Candida albicans (C. albicans)* strains. In addition to being flexible and adherent to the textile substrates, the nanocomposites exhibited remarkable microbial growth inhibitory effects.

## Introduction

The proliferation of microorganisms is a major concern for health organizations, whether it be medically speaking, in agriculture or simply in built environments. Improving the indoor air quality, while limiting the spread of bacteria, fungi, or viruses on various surfaces has become the focus of many research teams today, especially in the wake of the on-going COVID19 pandemic [1–3]. However, the overuse of antimicrobials since the 1950s has caused bacteria and fungi to develop strong antibiotic and antiseptic resistance [4–6]. Due to its versatility, nanotechnology has the potential of offering innovative, cost-effective and industrially viable solutions. Specifically, metal-based nanoparticles (MNPs) are of particular interest for such applications as they exhibit impressive antibacterial and antifungal properties. Unlike antibiotics for example, that target cell wall synthesis, translational machinery and DNA replication inside bacteria cells [7], MNPs simply attack the outer cell membrane and as such, are less likely to prompt resistance in microorganisms. In addition, their tunable sizes, shapes, and high surface area-to-mass ratio offer increased interactions with cells [8]. The prevalent MNPs used today as antimicrobial agents are copper [9] (or copper oxide [10]), gold [11–12], zinc oxide [13–14], and especially silver nanoparticles [15–18].

Silver is known to target peptidoglycane, a cellular membrane component of Gram-negative and -positive bacteria. If introduced directly in its ionic form, silver interacts with the electron-donor groups of the bacterial cell membrane [19–20], allowing its penetration inside the cytoplasm. This leads to the leakage of cellular components through the pores of the perforated cellular membrane. Once inside, the ions promote reactive oxygen species (ROS) generation, deactivate proteins, and block DNA replication [21]. Silver nanoparticles (AgNPs) have the added benefit of being ionic silver vessels, combining the latter’s antimicrobial properties with their own characteristics [16]. Pal et al. [22] demonstrated that triangular AgNPs seem to exhibit increased biocide activity compared to their spherical counterparts, while Martínez-Castañón et al. [23] stated that smaller AgNPs are more effective in penetrating the cell wall. AgNPs were also found to be efficient antifungal agents [24], in particular against the infectious *Candida albicans* yeast species [25].

The potential applications of AgNPs as antimicrobial agents can be further explored by combining them with a physical matrix. Textiles are a prime breeding ground for bacteria and fungi, given the right temperature, nutrient-available and humidity conditions. With this in mind, antimicrobial nanoparticles-functionalized textiles are being actively investigated [26], with specific emphasis on AgNPs [27]. Textiles have been successfully functionalized with AgNPs using a variety of both physical and chemical deposition techniques [28]. To name a few, Mei et al. [29] used magnetic sputtering to deposit AgNPs onto polyimide textiles; OhadiFar et al. [30] synthesized AgNPs on cotton fabrics using laser ablation, while Ahmad et al. [31] deposited AgNPs by the dip and dry method based on surface reduction reactions. However, the difference in expansion coefficients of the given metal layer and substrate can lead to surface defects under strain (cracks, loss of adhesion, etc.) [32–33]. The deposition techniques are also costly, time-consuming and restrictive (under vacuum, numerous steps, toxic chemicals, etc.), which limits industrial scale-up options.

Nanometal-polymer coatings offer an interesting alternative to the aforementioned metallized textiles. Such nanocomposites are prepared via ex situ or in situ approaches, in which MNPs are either synthesized beforehand and subsequently added to a polymer matrix or directly generated inside the polymer film. Ex situ methods include grafting [34], electrostatic [35] or polyol processes [36], but remain relatively difficult to implement and tend to cause NP self-aggregation. In situ methods are therefore generally preferred and typically require the polymer film surface to be treated with a metal precursor solution (layer-by-layer [37–38], sol–gel [39]) before undergoing thermal [40] or chemical reduction reactions [41].

The polymer coating adapts to various textile shapes, improves the adhesion between the MNPs and the substrate by compensating internal stresses and maintains the antimicrobial properties of the NPs. As the nanoparticles are embedded inside the polymer matrix, they are protected from external forces, which ultimately extends the lifetime of the functionalized textile. The antimicrobial properties of MNP-polymer composites have been extensively investigated [42–44] and research has gone into functionalizing textile fibers with the nanocomposites in situ [45–46]. Few studies seem to have been carried out on the coating of unaltered textile substrates with hybrid MNP-polymer films for antimicrobial applications.

In a previous work [47–48], we presented an innovative one-pot, one-step photoinduced synthesis to generate silver and gold-polymer nanofilms on a glass substrate. The kinetic coupling between the in situ chemical reduction of metallic precursors and photopolymerization of acrylic monomers ensures a depth-wise MNP distribution inside the cross-linked network, which prevents possible leaching processes. Based on these results, we investigated the antimicrobial properties (liquid and plate diffusion assays) of AgNP@polymer nanocomposites-coated textiles against *Escherichia coli* (*E. coli*) and *Candida albicans* (*C. albicans*) strains. The mechanical properties (flexibility, adhesion, abrasion) were also studied using a Mini-Martindale device, a standard scratch test kit, scanning electron microscopy (SEM), transmission electron microscopy (TEM), and reflectance measurements to assess the optical properties and the durability of the functionalized textiles.

## Results and Discussion

### Photoinduced synthesis of the Ag@polymer coating

**Figure 1:**
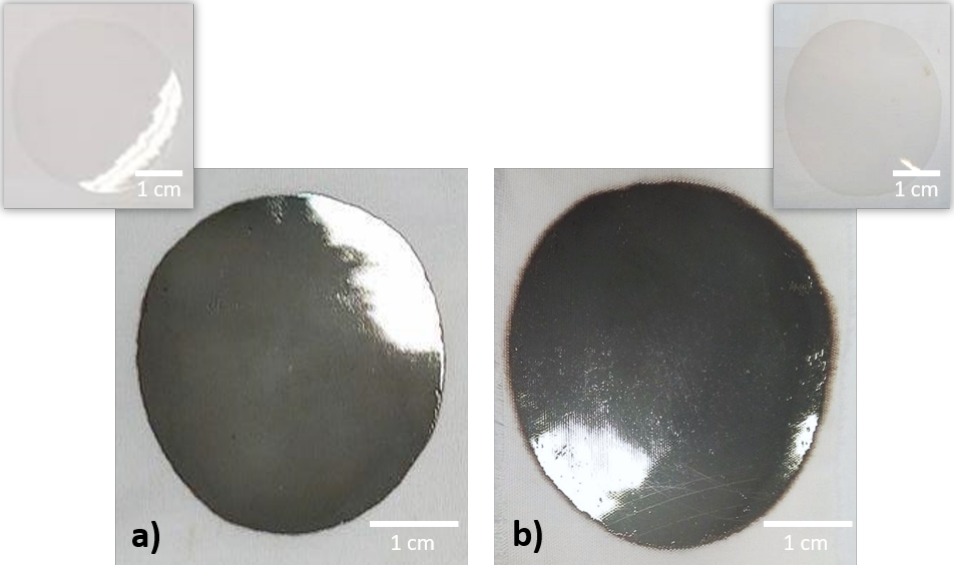
Cotton textile coated with Ag@PEG600DA (a), Ag@PEG600DA/PETIA (1:1) (b) and inserted images of PEG600DA and PEG600DA/PETIA polymers without AgNPs.

Specific monomers poly(ethylene glycol) 600 diacrylate (PEG600DA) and pentaerythritol triacrylate monomer (PETIA) used as comonomer (PEG600DA/PETIA with a 1:1 weight ratio) were mixed under magnetic stirring with diphenyl(2,4,6-trimethylbenzoyl)phosphine oxide (0.5 wt %) and the metal precursor AgNO_3_ (3 wt % and 5 wt %) for 1 h. After complete dissolution, this photosensitive formulation was applied to a cotton textile with an abyko-drive applicator (calibrated bar coater). The resulting smooth sample was then irradiated under UV light (600 mW/cm^2^) to obtain the final Ag@polymer textile material, with a coating thickness of 100 µm, measured with a Mitutoyo digital micrometer. Figure 1 shows images of the as-synthesized samples with and without AgNPs for the PEG600DA polymer and PEG600DA/PETIA copolymer matrixes coated onto cotton fabric.

### Optical and morphological properties

**Figure 2:**
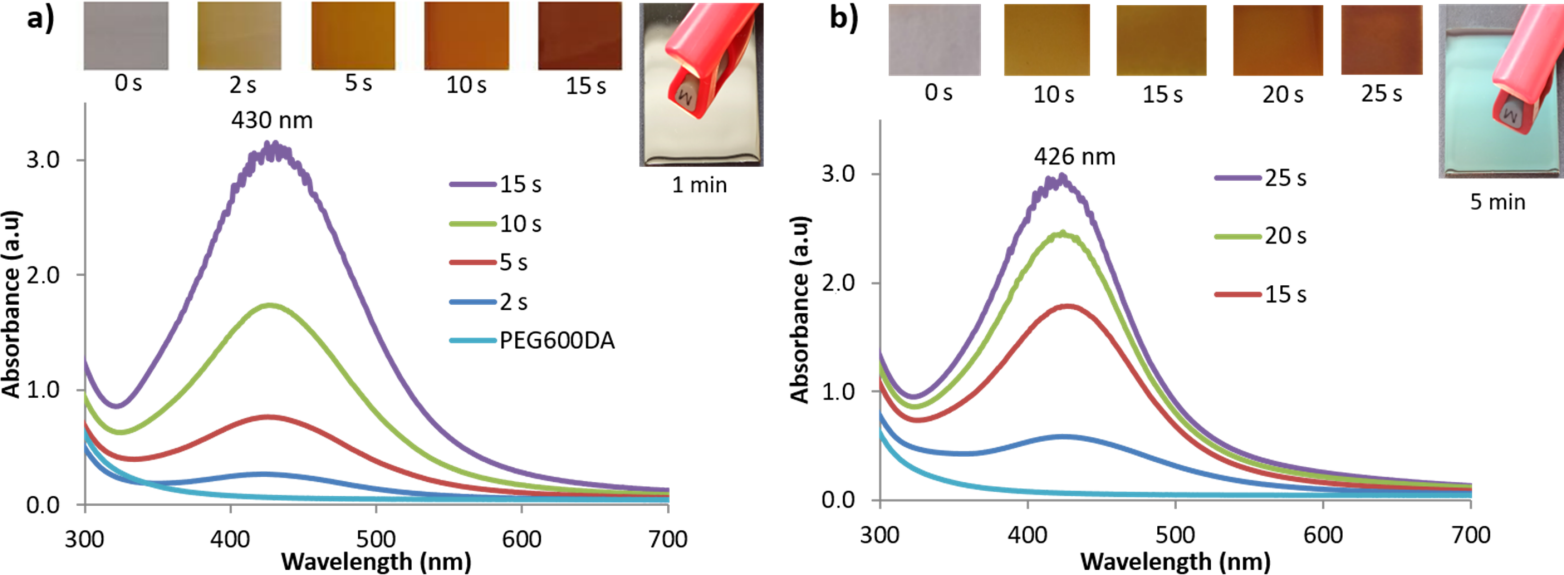
UV–vis spectroscopy monitoring of 100 µm-thick Ag@PEG600DA (a) and Ag@PEG600DA/PETIA (b) coatings, with images taken after increasing exposure times.

UV–vis spectroscopy was initially carried out on glass-coated Ag@PEG600DA or Ag@PEG600DA/PETIA samples, in order to follow the AgNP synthesis for different exposure times. The obtained spectra are presented in Figure 2, as well as images of the coatings taken for different exposure times. The characteristic surface plasmon resonance band of AgNPs for both coatings is observed around 430 nm and increases with exposure time, which indicates an increase in NP concentration. Since the absorption peak is 426 nm for the Ag@PEG600DA/PETIA coating, the particles are expected to be slightly smaller than those of the Ag@PEG600DA film. This can be explained by the fact that the PETIA comonomer is a triacrylate monomer, which increases the amount of cross-linked polymer chains compared to the diacrylate monomer (PEG600DA). Moreover, particle stabilization is ensured through interactions with the CO^•^ groups resulting from photoinitiator decomposition [49]. In addition, AgNP interactions with the polymer’s non-bonding doublets of oxygen atoms create steric hindrance and avoid their aggregation. The coating, colorless before irradiation, becomes progressively pale yellow, orange, then finally brown. Due to the strong increase of AgNPs, the measurement system reaches the detection limit (OD > 3) after 15 seconds of exposure in the case of the Ag@PEG600DA coating (Figure 2a). As can be seen in Figure 2, AgNP synthesis is much faster in Ag@PEG600DA coatings than in the case of the PEG600DA/PETIA matrix. Indeed, as a diacrylate, PEG600DA offers less cross-linking sites than PETIA, which promotes nanoparticle formation and coalescence during UV exposure. When comparing the absorbance spectra for both coatings after 15 s irradiation, the full-widths at half maximum (FMWH) are calculated to be 134 and 131 nm, for Ag@PEG600DA and Ag@PEG600DA/PETIA, respectively (Figure 2c). Consequently, the nanoparticle size dispersion can be expected to be relatively similar for both coatings with a slightly smaller size dispersion for the Ag@PEG600DA/PETIA coating. Further exposure to UV light eventually turns the surface of the coating into a metallic silver layer with remarkable mirror-like properties (Figure 2).

**Figure 3:**
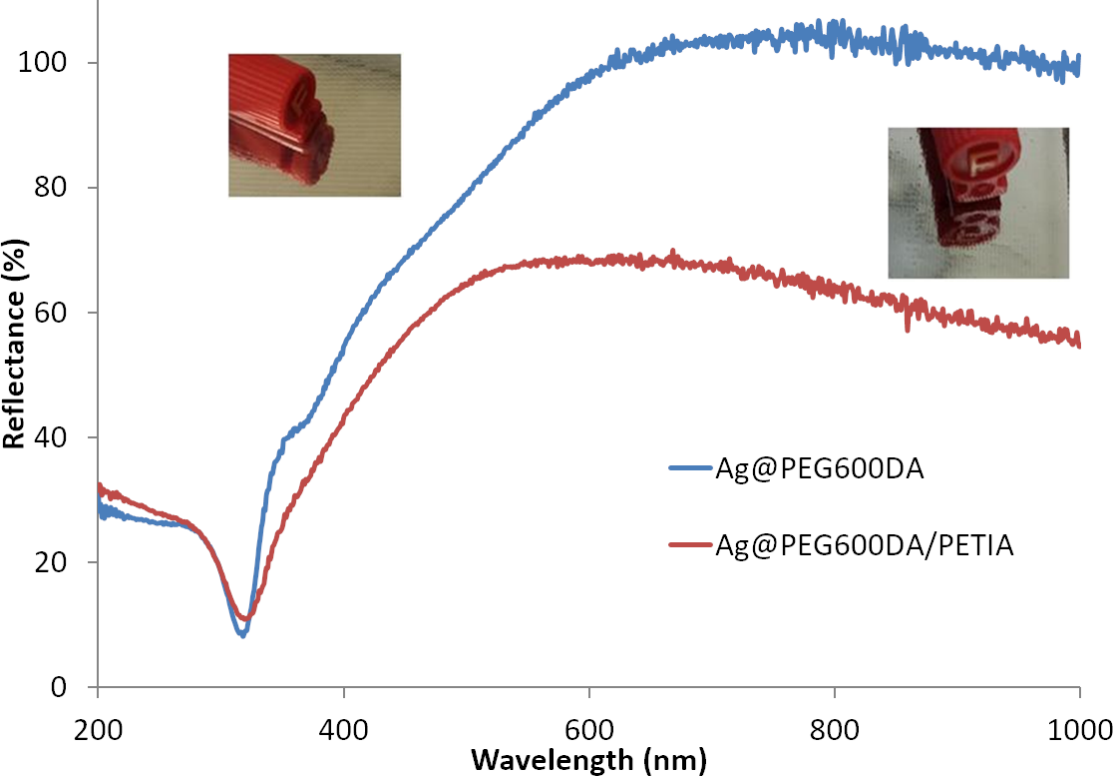
Reflectance measurements and corresponding images of Ag@polymer (PEG600DA and PEG600DA/PETIA) functionalized textiles.

The optical properties exhibited by the Ag@polymer-functionalized textiles (Figure 1a and 1b) were then investigated via reflectance measurements. As shown in Figure 3, the Ag@PEG600DA coating is more reflective than the Ag@PEG600DA/PETIA sample (97% and 68% at 650 nm, respectively).

**Figure 4:**
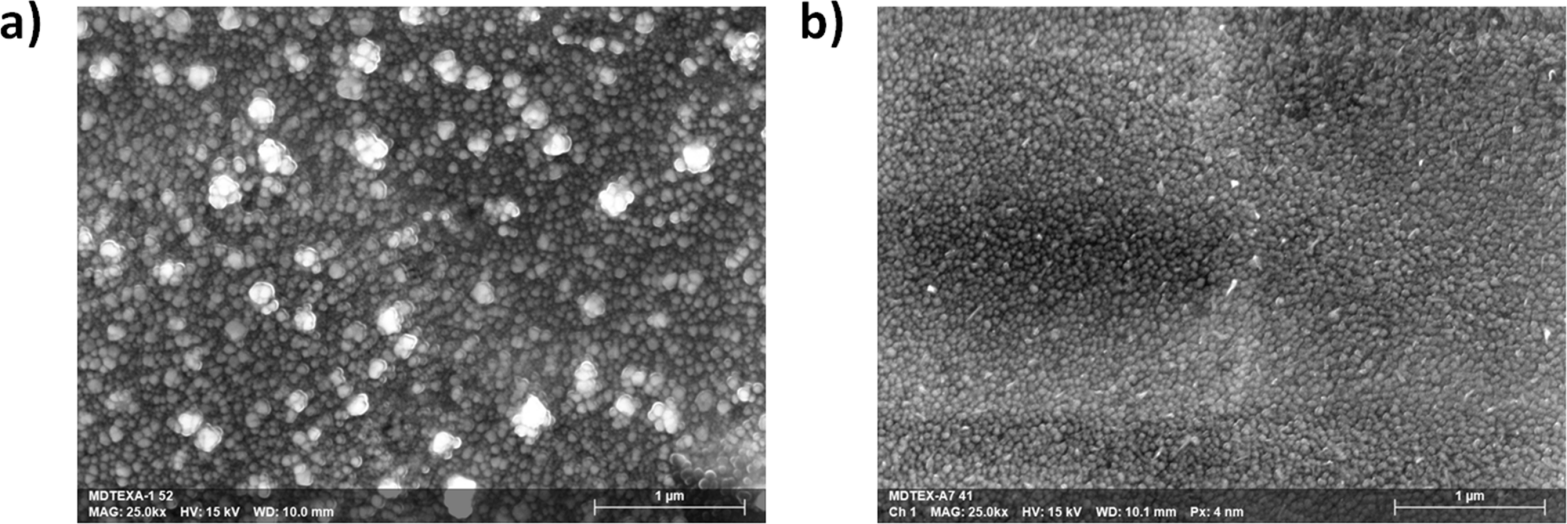
SEM of the 100 µm-thick Ag@PEG600DA (a) and Ag@PEG600DA/PETIA (b) coatings.

Particle assembly and distribution, as well as the final thickness of the metallic layer, account for this difference in reflectivity. Scanning electron microscopy (SEM) carried out on the surface of functionalized textiles revealed the homogenous distribution of AgNPs, with average sizes of 62 ± 2 nm and 58 ± 1 nm for the Ag@PEG600DA (Figure 4a) and Ag@PEG600DA/PETIA (Figure 4b) coatings, respectively. The AgNP size dispersion is also slightly higher in the case of Ag@PEG600DA. Both results are coherent with the peak and FMWH values calculated from the absorbance spectra (Figure 2c).

**Figure 5:**
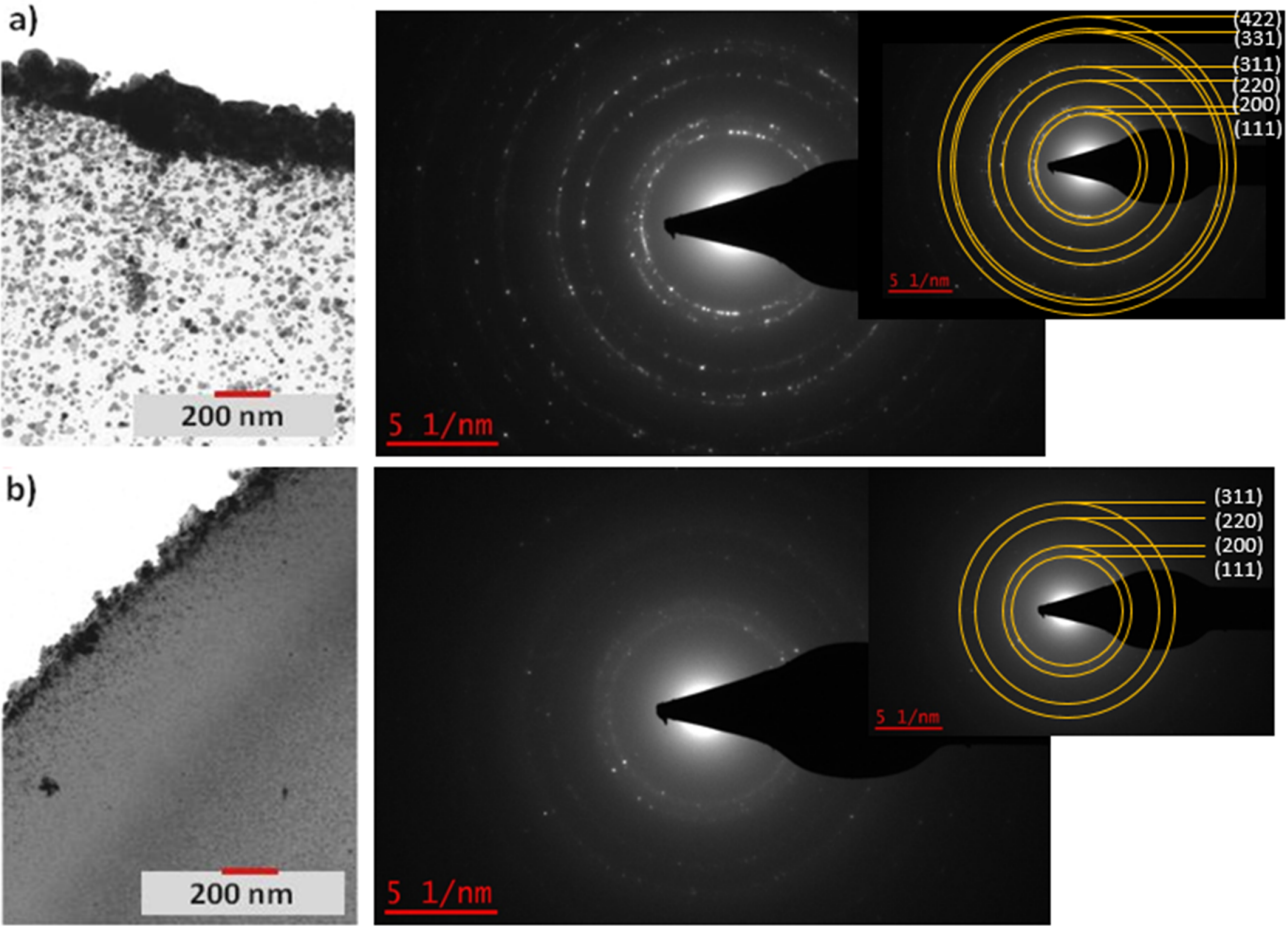
TEM cross sections of the Ag@PEG600DA (a) and the Ag@PEG600DA/PETIA (b) with the selected area electron diffraction (SAED) patterns.

Transmission electron microscopy (TEM) cross sections of 100 µm-thick samples were then taken for both functionalized textiles (Figure 5). In both cases, a gradient distribution of AgNPs was observed after UV synthesis, with concentrations increasing from the depth to the surface of the sample. The top metal layer thickness of the Ag@PEG600DA film reached 250 nm (Figure 5a), compared to 90 nm for the Ag@PEG600DA/PETIA coating (Figure 5b). AgNPs trapped inside the polymers have sizes of 10–30 nm in the case of PEG600DA, while barely reaching 10 nm for PEG600DA/PETIA. Since the PETIA monomer has three reactive groups, the synthesized polymer network is denser than in the case of PEG600DA diacrylate monomer, which ultimately limits not only the increase in particle size but also the migration/diffusion of silver ions and NPs towards the surface. This affects the final thickness of the top metal layer, the depth of the concentration gradient and the final size of the AgNPs (see Figure 5). As a result, high reflectance is linked to the formation of a thick and compact top metal layer, which is indeed the case for the Ag@PEG600DA coating.

Moreover, the selected area electron diffraction (SAED) patterns obtained during TEM analysis proved the high crystallinity of the synthesized NPs and the diffraction rings which indicate the polycrystalline nature of the AgNPs have been indexed to the (111), (200), (220) and (311) planes and correspond only to the face-centered cubic (fcc) crystal structure of metallic silver (JCPDS, No. 04-0783) (Figure 5). This result is of great importance because it confirms the complete photoreduction of the precursor and the absence of any oxidized form of silver in the Ag@polymer coating.

### Mechanical properties

#### Flexibility and adhesion

**Figure 6:**
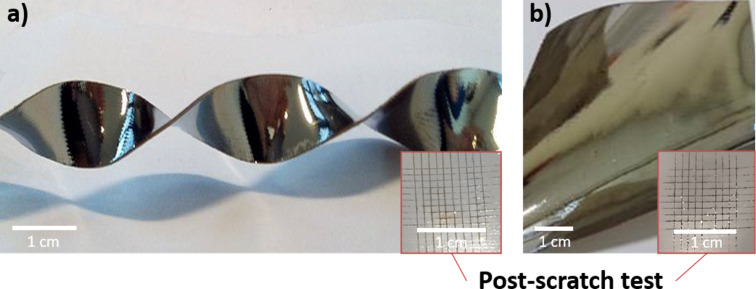
Images of flexible Ag@PEG600DA (a) and Ag@PEG600DA/PETIA (b) samples; inserted post-scratch tests.

Functionalization of the textiles with the Ag@polymer does not significantly affect the flexibility of the original cotton substrate (Figure 6). In the case of Ag@PEG600DA coatings, the samples do not show any cracking or loss of adhesion at the coating/textile interface following a bending-type deformation for example; the metal surface remains visually intact. However, the use of the PEG600DA/PETIA polymer matrix leads to an increase in rigidity, linked to the trifunctional monomer architecture, which favors polymer chain cross-linking. As such, the Ag@PEG600DA/PETIA coatings are rendered more sensitive to this type of deformation, when pressure is applied to the functionalized side of the fabric.

In order to assess the adhesion properties of the Ag@polymer coatings on the cotton fabric, scratch tests were carried out on the smooth surfaces of each type of coating, i.e., Ag@PEG600DA and Ag@PEG600DA/PETIA. In accordance with the NF EN ISO 2409 standard regarding paints and varnishes, a scratch test was performed by first creating a grid (1 mm evenly spaced incisions) in the surface of the coating. Then a standard-approved 8.75 N adhesive tape was applied and taken off. Depending on the amount of coating remaining after the scratch test, a value of 0 to 5 is attributed, 0 corresponding to perfect adhesion. Figure 6 shows the results for both the Ag@PEG600DA (Figure 6a) and Ag@PEG600DA/PETIA (Figure 6b) coatings.

In both cases, excellent adhesion was observed (value of 0), which can be explained by the remarkable textile wetting properties of the photosensitive formulation. The choice of polymer matrix effectively dictates the observed wettability and adhesion to the chosen substrate. The adhesion properties can also be explained by the innovative structure of the depth-wise gradient distribution of AgNPs in the metallic coatings. Indeed, as the AgNPs and the polymer matrix are synthesized simultaneously, the polymer chains are able to insinuate themselves between the particles, effectively anchoring them to the rest of the coating.

#### Abrasion

**Figure 7:**
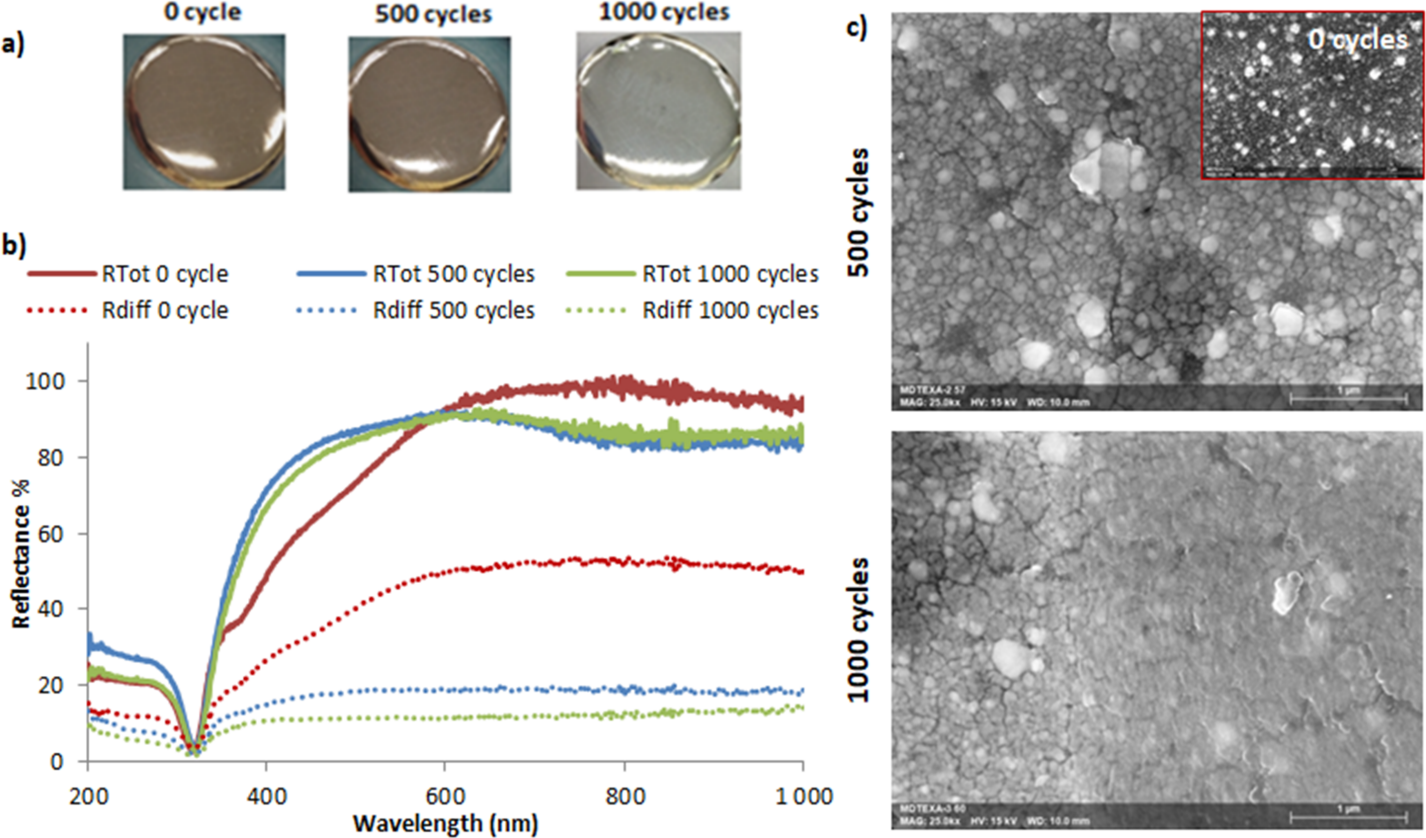
Influence of 500 and 1000 abrasion cycles on the surface of an Ag@PEG600DA coating (a) and on the total and diffuse reflectance (b). Associated SEM surface images (c).

The longevity of the functionalized textiles was next assessed by testing the abrasion resistance of the coatings, with a particular emphasis on the optical properties of the top metal layer. Circular samples (3.8 cm in diameter) underwent the Martindale test (ISO 12947 standard), which imitates the natural deterioration of a material with time. Throughout the test, the samples sustain a fixed number of abrasion cycles, during which an abrasive material (wool here) is frictioned linearly on the surface with a force of 12 N, following a Lissajous curves pattern. Figure 7a shows the Ag@PEG600DA-functionalized textile before and after 500 and 1000 abrasion cycles. The total reflectance spectra and its diffuse component are also presented (Figure 7b). Before abrasion, the Ag@PEG600DA coating is highly reflective, with a total reflectance value of 97% at 700 nm, of which 52% comes from the diffuse component. The applied friction seems to polish the surface, increasing its mirror-like aspect. After 500 cycles, the total reflectance increased significantly between 400 and 580 nm, while dropping slightly (10%) in the 580–1000 nm range. The diffuse reflectance effectively decreased from 52 to 20% at 700 nm.

Indeed, friction causes a localized rise in temperature between particles, which promotes a reduction in surface roughness, as can be seen in the SEM images (Figure 7c). Consequently, the diffuse reflectivity drops in favor of the specular reflectance. The particles are no longer simply juxtaposed but form a continuous silver layer, especially after 1000 friction cycles. The characteristic silver plasmon band, identifiable between 380 and 500 nm on the pre-abrasion reflectance spectra, has effectively disappeared and the resulting curve resembles that of bulk silver.

**Figure 8:**
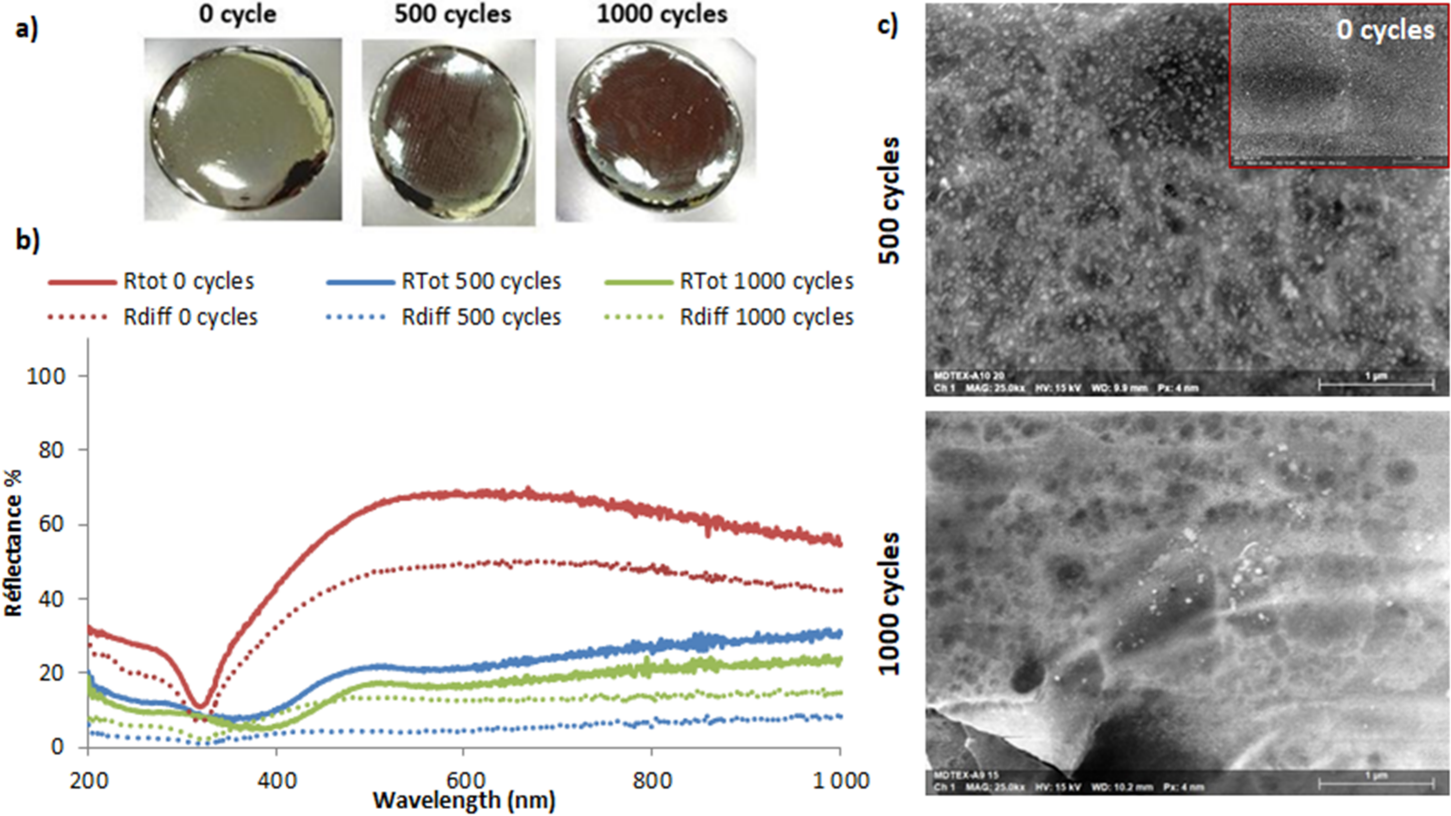
Influence of 500 and 1000 abrasion cycles on the surface of an Ag@PEG600DA/PETIA coating (1:1) (a) and on the total and diffuse reflectance (b). Associated SEM surface images (c).

Regarding the Ag@PEG600DA/PETIA coating, surface images and reflectance spectra of the samples pre- and post-abrasion are shown in Figure 8a and 8b. Interestingly, the total reflectance is lower than that of the Ag@PEG600DA coating (68% compared to 95% at 600 nm). After 500 abrasion cycles, the metallic layer was damaged, even more so after 1000 cycles, as can be seen in Figure 8a. This deterioration causes a significant drop in reflectance, mainly diffuse. The underlying metallic layer appears brown and exhibits an absorption band at 430 nm, linked to the plasmon resonance of AgNPs (Figure 8c), trapped inside the polymer matrix.

To better understand this sudden decrease in reflectance, the surface was characterized by SEM (Figure 8c). Before the abrasion test, the surface was completely covered with homogenously distributed and almost monodispersed nanoparticles (25–50 nm). Post-abrasion, only a few nanoparticles remain visible, which corroborates the resurgence of the AgNP plasmon band.

Unsurprisingly, the nature of the polymer matrix affects the resistance of the final material to abrasion. The rigid nature of the PETIA monomer could account for the low abrasion resistance and decrease in reflectance of the Ag@PEG600DA/PETIA coating, compared to that of Ag@PEG600DA. As previously explained, the PETIA monomer prevents the easy diffusion of silver ions towards the surface, resulting in the observed thin top metal layer (90 nm) of the Ag@PEG600DA/PETIA film, as confirmed in the TEM images (Figure 5). The metal layer is not thick enough to withstand the damage inflicted by the abrasion process.

#### Rheological properties

**Figure 9:**
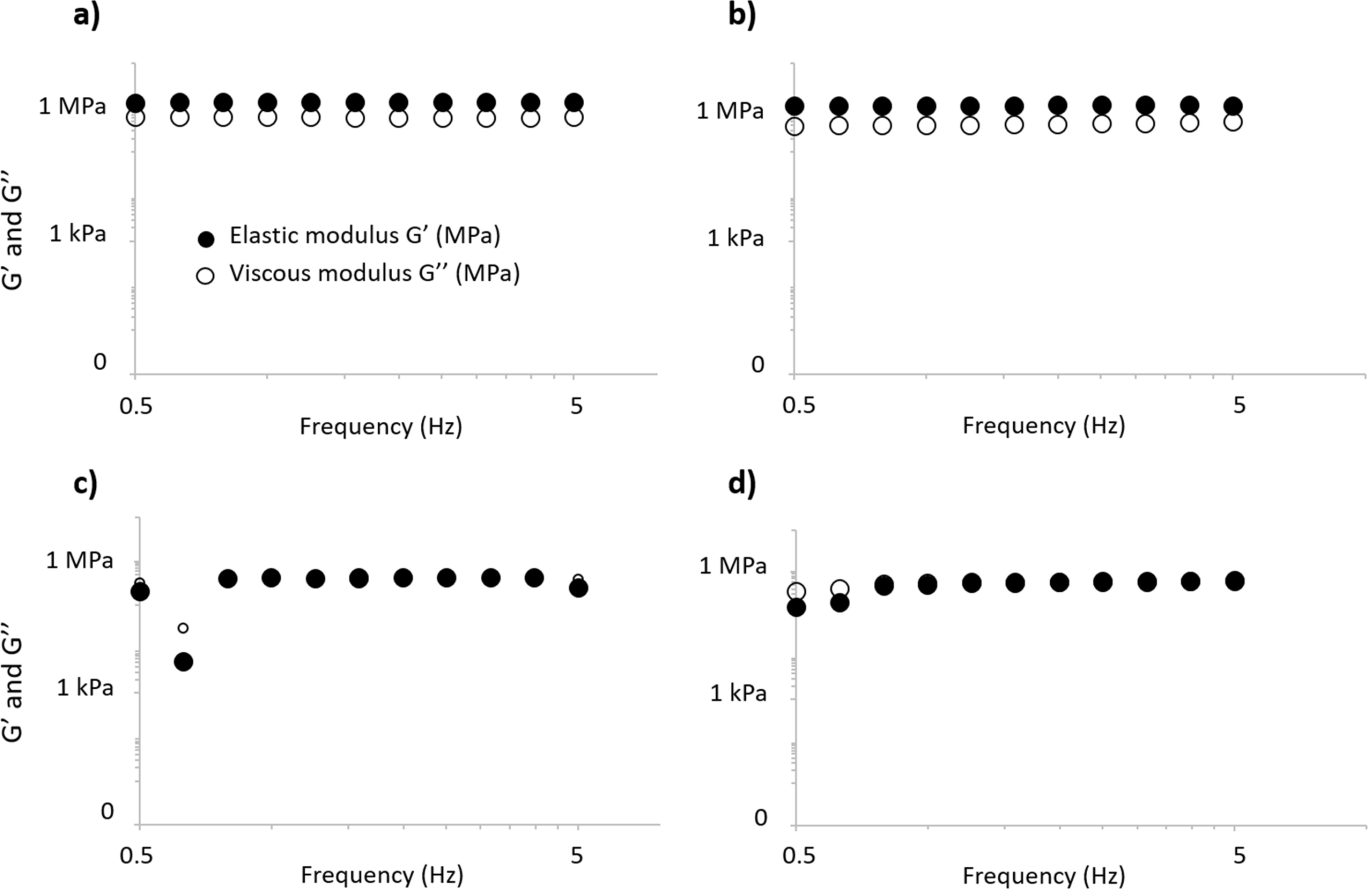
Rheological characteristics of uncoated textile (a), PEG600DA/PETIA (b) and Ag@PEG600DA/PETIA (3 and 5 wt %) (c and d, respectively)-coated textiles in the 0.5–5 Hz frequency range.

Potential significant changes in the viscoelastic properties of the functionalized material were suspected of being induced by the addition of AgNPs in the polymer matrix, which was assayed by rheometry measurements. This analysis confirms that coatings with and without Ag exhibit a viscoelastic behavior (Figure 9a–d). The storage modulus G’ is not significantly affected by the polymer matrix, but changes with the presence of Ag in the coating. Both storage moduli of uncoated and PEG600DA/PETIA-coated textiles are about 1 MPa (1.1 ± 0.1 MPa and 1.0 ± 0.1 MPa, respectively), whereas they are 0.3 ± 0.1 MPa and 0.6 ± 0.1 MPa for Ag@PEG600DA/PETIA 3 and 5 wt % coatings, respectively. This decrease in the elastic component may be due to the presence of AgNPs in the polymer matrix. In addition, their general viscoelastic behaviors differ: PEG600DA/PETIA-coated textile has a predominant elastic behavior in the tested frequency range (G’/G” > 1), while Ag@PEG600DA (3 and 5 wt %) coatings display similar levels in viscous and elastic behaviors in the same frequency range (Figure 9b). This appears to result from the decrease in the elastic component rather than from a viscosity modification, which may be due to the textile. These changes are not expected to negatively impact the use of the functionalized textile; rather, the improvement of the elastic properties induced by addition of Ag may be beneficial for elongation properties without any permanent, undesirable deformation.

#### Antimicrobial properties

The antimicrobial activity of the functionalized textiles was quantified via a liquid diffusion assay, while a plate diffusion assay was used to visualize the microbial growth inhibition. Preliminary tests showed that the Ag@PEG600DA-coated textile does not stay flat during immersion into microbial suspensions, despite being glued to the glass dishes. For this reason, antimicrobial characterizations were only carried out on Ag@PEG600DA/PETIA loaded with 3 wt % and 5 wt % of AgNO_3_ in order to observe the impact of the silver concentration on the *E. coli* bacteria and the *C. albicans* fungus.

#### Liquid diffusion assay

**Figure 10:**
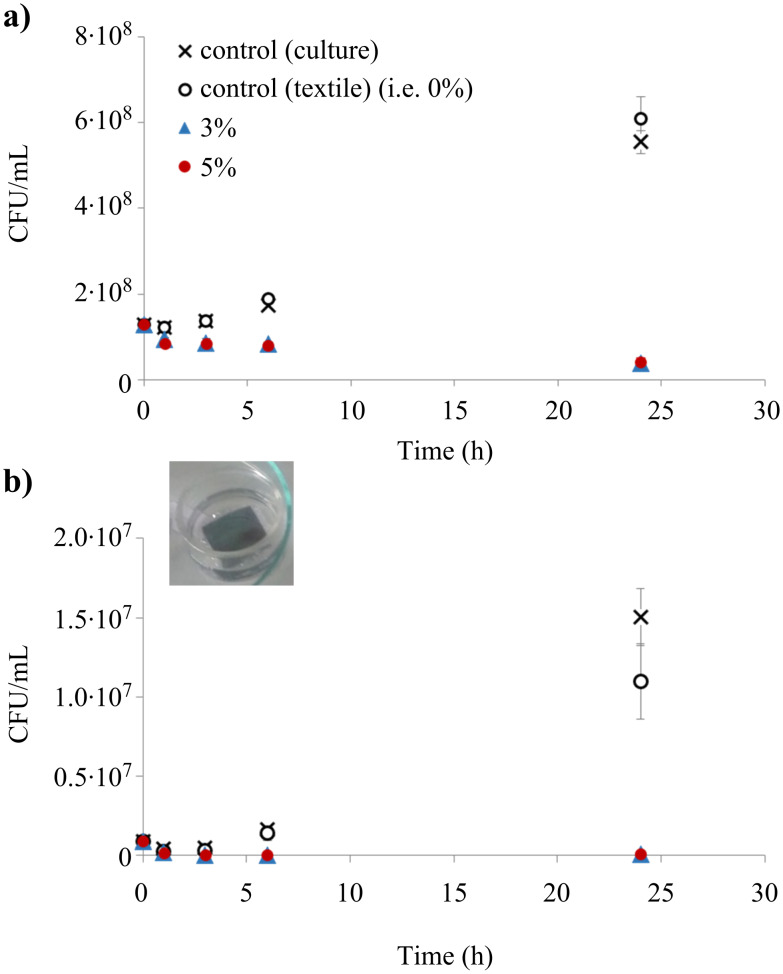
Colony forming units (CFU) per mL of suspension calculated from OD_600nm_ measurements of *E. coli* (a) or *C. albicans* (b) cultures after 0, 1, 3, 6, or 24 hours contact with the samples; inserted image of immersed functionalized textile. For both microorganisms, 3 wt % and 5 wt % conditions are significantly different (*p*-value < 0.05) from the controls whatever the time (except at time = 0, i.e., inoculation suspension, and at 1 h for *C. albicans*). The 3 wt % and 5 wt % conditions are not significantly different from each other. Controls are not significantly different from each other, except at 24 h of culture for *C. albicans*.

Attenuance measurements (OD_600_, i.e., OD at 600 nm), performed on microbial suspensions in contact with the functionalized textiles after 1, 3, 6, and 24 h of immersion, are plotted in Figure 10. For both *E. coli* and *C. albicans* microorganisms, control cultures (without any textile or with silver-free, functionalized textile) show an increase of CFU/mL with sample immersion time, in agreement with growth rates expected in the M63G medium [50]. Contrarily, Ag@PEG600DA/PETIA samples indicate a decrease of CFU/mL values, thus reflecting a lower cell concentration in this suspension than in those cultivated without the sample. This effect can be attributed to the presence of silver, since microbial growth is also observed in the case of the control material (cotton coated with the Ag-free PEG600DA/PETIA polymer matrix). Nevertheless, this growth is lesser than that of the control culture for the *C. albicans* microorganism, suggesting that the polymer matrix releases one or more compounds capable of inhibiting fungal growth. In a previous study [51], we demonstrated that the polymer matrix is capable of releasing several organic compounds due to photodegradation, including formic acid. This weak acid has been shown to exhibit antifungal properties. Lastauskiené et al. [52] showed that formic acid can induce programmed cell death in several Candida species, including *C. albicans* pathogens. In vitro testing revealed MIC values of 1.22 mg/mL (26.5 mM) on *C. albicans* thus suggesting a mild but significant fungicidal effect. This is similar to the efficacy of acetic acid and can be classified as slightly fungicidal [53–54].

**Figure 11:**
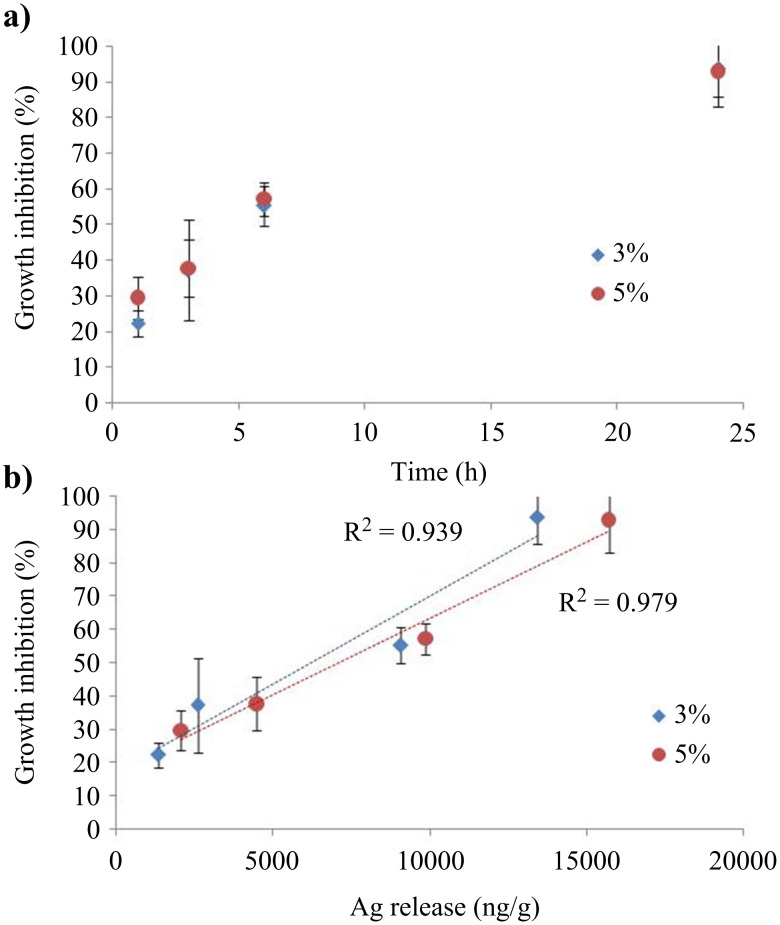
Growth inhibition of bacteria (*E. coli*) for different sample immersion times (a), and for the corresponding amount of released silver (b).

The inhibition rate of microorganism growth was determined from the OD_600nm_ measurements, for suspensions in contact with 3 wt % and 5 wt % AgNO_3_-loaded samples. The results are presented in Figure 11a for *E. coli* and remarkably a total inhibition of growth was reached within 24 hours. The silver concentration has no significant impact on antibacterial activity, which indicates that a mere 3 wt % Ag-loaded sample is sufficient to obtain complete elimination of bacteria. The released silver quantity was determined by ICP analysis and has been correlated with the bacteria growth inhibition rate (Figure 11b). For equal immersion times in microbial suspensions, the amount of silver released is more important for the 5 wt %-loaded samples, but in both cases (3 wt % and 5 wt %) a linear evolution is observed between the growth inhibition and the released silver content. A complete inhibition was achieved for a silver quantity of 15 µg/g released in liquid media. As demonstrated by Hsueh et al. [55] AgNPs can be considered as containers continuously releasing silver ions, thus indirectly avoiding planktonic bacteria proliferation by the direct action of silver ions. The capacity of 3 wt % and 5 wt %-loaded textiles to inhibit the growth of *E. coli* is thought to be related to this mechanism.

**Figure 12:**
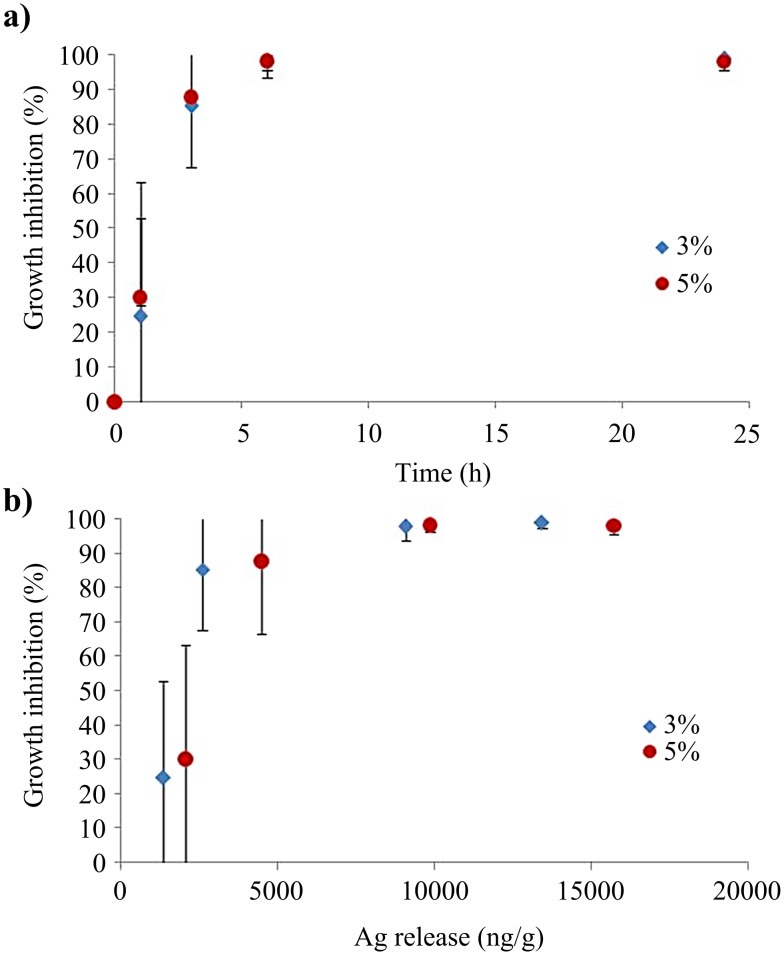
Growth inhibition of fungus (*C. albicans*) for different sample immersion times (a), and for the corresponding amount of released silver (b).

Concerning the fungus, similarly to bacteria, the amount of loaded silver does not have a significant impact on the antifungal activity (Figure 12a). Nevertheless, the antifungal activity of the Ag@PEG600DA/PETIA coating (3 and 5 wt %) results in total inhibition of fungal growth within 6 hours. The silver amount, released immediately after sample immersion, is sufficient in blocking *C. albicans* growth (Figure 12b). It should be noted that the required silver quantity to effectively inhibit microorganism growth is lower for *C. albicans* fungus (≈10 µg/g) than for *E. coli* bacteria (≈15 µg/g), which enables a faster growth inhibition of the fungi. In addition, the response of *C. albicans* to doses lower than the maximum effective dose is not linear, unlike that of *E. coli* bacteria, confirming a higher sensitivity to silver, even in the presence of low amounts of released silver (90% inhibition rate for approximately 2.5 µg/g released silver).

This effect, more pronounced for *C. albicans* than *E. coli* has also been observed by Akinsiku et al. [56], who determined that the sensitivity to silver varies by a factor of 5 between clinical isolates of *E. coli* and *C. albicans*. The mechanisms leading to this difference do not seem to have been elucidated so far. However, some hypotheses can be put forward. In the present study, the antimicrobial activity of *E. coli* and *C. albicans* is only due to the action of silver ions released by AgNPs [20]. Ionic silver first perforates the cell wall, enters the microorganism and eventually induces cell apoptosis. Indeed, once inside the cell, silver interacts with compounds generating reactive oxygen species (ROS) or blocks DNA replication and protein action (enzymes), thereby leading to cell death. The increased growth inhibition observed for *C. albicans* compared to *E. coli* could be explained in part by the difference in kinetics of silver penetration into microorganisms, possibly due to the significant difference in the external cell wall composition. Peptidoglycan is a component of bacterial cell wall that was suggested as the main target of silver [57]. In *E. coli* and the other Gram-negative bacteria, the peptidoglycan layer is protected by an outer membrane, which may limit the interactions with silver in comparison to Gram-positive bacteria, that exhibit a peptidoglycan layer directly in contact with the surrounding medium. Similar to Gram-positive bacteria, *C. albicans* exhibits an external cell wall, in direct contact with the surrounding medium, which also contains *N*-acetylglucosamine, one of the two main components of peptidoglycan, and may therefore be a target for silver [58]. In addition, silver has been shown to target several fatty acids of the membrane of *C. albicans*, which is thought to affect the hyphal morphogenesis of yeast, a vital process of the cell [59]. Moreover, *C. albicans* has been reported as particularly sensitive to ROS, which may be one of the reasons for its higher sensitivity to silver than other microorganisms [60].

The culture medium could also impact microorganism susceptibility to silver ions. For comparison purposes, the same medium was chosen for both bacteria and fungus. M63G medium has been preferred since it does not reduce silver antimicrobial activity contrary to media containing components such as Cl^−^ or other biomolecules, which interact with ionic silver [20]. In addition, it provides nutrient conditions more likely to mimic real conditions of use of the functionalized textiles developed in this study than rich nutritive media that supply microorganisms with an unusual, easy access to large amounts of nutrients. M63G is known to offer suitable conditions for *E. coli* growth [41] and it was shown to also allow *C. albicans* growth in a preliminary study (data not shown). Of course, *E. coli* and *C. albicans* growths are limited in M63G compared to that in the LB and Sabouraud nutritive media that are perfectly adapted to these microorganisms, respectively. In addition, physiological changes induced by a different access to nutrient and other compounts are expected to impact the microbial susceptibility to antimicrobials including silver [61]. They may lead to reduced, increased, or even unaltered minimal inhibitory concentrations, but the exact changes are unknown. In this study, finally, even if a direct impact of the M63G environment on microorganism susceptibility is plausible, it is not currently possible to confirm that the culture medium played a significant role in the observed difference of inhibition kinetics.

#### Plate diffusion assay

**Figure 13:**
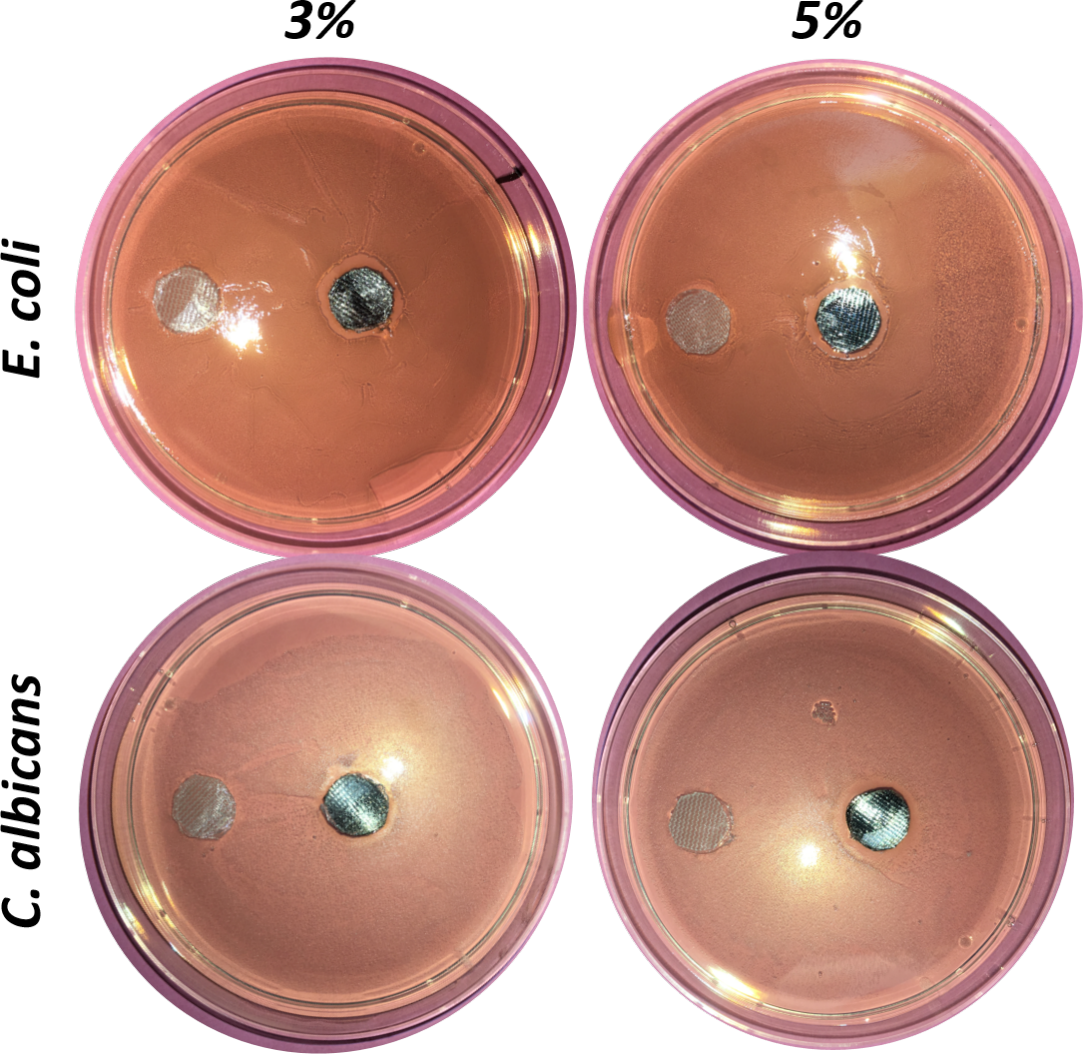
Plate diffusion test for the observation of growth inhibition zones of *E. coli* and *C. albicans* after 24 h incubation.

Functionalized and uncoated textiles were placed on a microbial film and the resulting microbial growth around the samples was examined to identify the potential presence of a growth inhibition zone. This study provides visual information from which quantitative trends could be extracted. The results obtained after 24 h incubation, at 30 °C for *E. coli* and 37 °C for *C. albicans*, are presented in Figure 13 and Table 1. For *E. coli*, a growth inhibition zone is clearly noticeable around the silver-loaded samples and increases with silver concentration. The control sample (cotton coated with the PEG600DA/PETIA polymer matrix) exhibits a slight inhibition zone. Concerning fungus, the control sample shows little change but an inhibition zone was clearly observed around the silver-loaded samples. The antimicrobial activities on *E. coli* and *C. albicans*, confirmed by quantitative data reported in Table 1, can both be attributed to silver ions released from the functionalized materials, as explained above for assays in liquid. As experiment replications show important variation, no conclusive information could be extracted from the differences noticed between *E. coli* and *C. albicans* or between 3 wt % and 5 wt % Ag-loaded samples. Finally, the plate diffusion assay demonstrates lower antimicrobial activity than the liquid diffusion tests. This can be first explained by silver’s limited diffusion through agar, compared to liquid media. In addition, the sample surface contribution is limited to its immediate vicinity in the case of the plate diffusion test, whereas the whole surface contributes in liquid tests. In other words, regarding the surrounding medium, the functionalized textile can protect its surface and immediate vicinity without unwished release in the environment, or provide a protective inhibitory effect in the whole liquid surrounding.

**Table 1:** Growth inhibition zone sizes for *E. coli* and *C. albicans*. Both total (i.e., sample and inhibition zones together) and sample areas are measured by image analysis with the diameter of the Petri dish as the distance reference). Neat inhibition area was defined as the difference between total and sample areas. The 3 wt % and 5 wt % conditions are significantly different from the controls (*p*-value < 0.05) for both microorganisms and for *E. coli* only, respectively. The 3 wt % and 5 wt % conditions are not significantly different from each other. Samples are not significantly different from each other.

	Surface (mm^2^)	Polymer	3 wt %	5 wt %
		
microorganism	mean sample surface	112 ± 7		

*E .coli*	sample + inhibition zone	140 ± 19	218 ± 40	185 ± 25
	neat inhibition zone	31 ± 15	109 ± 40	75 ± 25
*C. albicans*	total inhibition zone	115 ± 17	133 ± 10	179 ± 26
	neat inhibition zone	8 ± 19	16 ± 14	68 ± 0

The antimicrobial activity of the Ag@PEG600DA/PETIA coating was thus confirmed for both *E. coli* and *C. albicans*. As observed during the abrasion tests, this sample exhibits a thinner silver top layer (90 nm) at the sample surface than the Ag@PEG600DA coating (250 nm), suggesting the latter would present superior antimicrobial activity due to the increased amount of available silver.

## Conclusion

The innovative photoinduced approach led to the successful functionalization of nano-architectured Ag@polymer composites onto textile substrates. Two different biocompatible polymer matrixes were compared in terms of AgNP growth, overall mechanical properties and behavior in biological environments. The NPs@polymer-coated textiles demonstrated very interesting optical and mechanical properties linked to the depth-wise gradient distribution of AgNPs in the metallic coatings. The choice of polymer matrix strongly affects the reflectivity and mechanical longevity of the synthesized films. The PETIA monomer increases the degree of cross-linked polymer chains, which limits the diffusion of AgNPs towards the surface of the coating. This accounts for the slightly lower reflectivity and resistance to abrasion of Ag@PEG600DA/PETIA coatings compared to Ag@PEG600DA ones. Due to its remarkable flexibility, PEG600DA on its own facilitates AgNP formation but has difficulty maintaining its shape in a biological medium, hence the choice of a second more rigid PEG600DA/PETIA copolymer matrix. The functionalized textiles exhibited impressive antimicrobial properties when loaded with 3 and 5 wt % Ag and tested against *E. coli* and *C. albicans* strains. In both liquid and agar media, low amounts of released ionic silver were sufficient in inhibiting the growth of bacteria and fungi, *C. albicans* showing a higher sensitivity than *E. coli* in liquid media (complete inhibition for approximately 2.5 and 15 µg/g released silver, respectively). The nanocomposite structure can thus be tuned in order to provide the best compromise between antimicrobial activity and mechanical longevity depending on the field of application.

## Experimental

### Materials

Silver nitrate (AgNO_3_, 99%) and diphenyl(2,4,6-trimethylbenzoyl)phosphine oxide were purchased from Sigma-Aldrich and the two monomers pentaerythritol triacrylate monomer (PETIA) and poly(ethylene glycol) 600 diacrylate (PEG600DA) were puchased from Sartomer Company. Inc. All products were used as received. A simple cotton textile was used as a substrate.

### Microbial strains and culture methods

Microbiological assays were conducted with a non-pathogenic, published bacteria strain, *Escherichia coli* (*E. coli*) SCC1 [62], and a yeast strain, *Candida albicans* (*C. albicans*) 1602m 280057, isolated from an infected patient. Microbial cells, previously frozen at −80 °C, were spread on LB (Lysogeny broth, Sigma-Aldrich) or Sabouraud (COGER) agar plates and cultured for two nights at 30 °C for *E. coli* and 37 °C for *C. albicans*, respectively. Pre-cultures of *E. coli* in LB and *C. albicans* in M63G medium (a *E. coli*-selective composed of 0.1 M KH_2_PO_4_, 20 wt % (NH_4_)_2_SO_4_, 0.1 wt % FeSO_4_, 20 wt % MgSO_4_, 6 M KOH, 0.05 wt % vitamin B1 and 10 wt % glucose; pH adjusted at 6.8; all products purchased by Sigma-Aldrich) were prepared [41], before undergoing an overnight incubation at 30 °C and 37 °C, respectively. The as-prepared pre-cultures were then used for the plate diffusion assays.

Further cultures were prepared for the liquid diffusion assay using 10% of the pre-culture volumes in fresh M63G medium for *E. coli* and *C. albicans*. They were incubated for 4 h at 30 °C and 37 °C, respectively, before harvesting the microbial cells by centrifugation. Harvested bacteria pellets were resuspended in fresh M63G medium and the as-obtained bacterial suspensions were adjusted by dilution to an optical density at 600 nm (OD_600_) of 0.1 (2.9 × 10^7^ CFU/mL of *E. coli* and 5 × 10^5^ CFU/mL of *C. albicans*).

### Characterization techniques

UV irradiations were carried out using a LLC UV lamp from Heraeus Noblelight America with H bulb reflector, delivering an actinic beam centered on 365 nm. Optical properties were evaluated with an Evolution E200 UV–vis spectrophotometer from Thermo Fisher Scientific, equipped with an integration sphere for reflectance measurements. A Bio photometer UV–vis spectrometer from Eppendorf was used to assess bacteria and yeast suspensions by OD measurements at 600 nm.

Surface characterizations were carried out using transmission electron microscopy (TEM) at 200 kV on a Philips CM200 instrument (LaB6 cathode) and scanning electron microscopy (SEM) on a JEOL IT800SHL scanning electron microscope with a magnification of 100000. Each microscope was equipped with a software capable of measuring particle diameters. Abrasion tests were performed with a Mini Martindale device, applying 12 N on the samples for 500 and 1000 cycles. A rotating KinexusUltra rheometer (Malvern, Great Britain) was used to perform rheometry measurements in dry conditions (25 °C). A plate-plate geometry (20 mm parallel plates) was set up. PEG600DA and Ag@PEG600DA disks of 20 mm in diameter were compressed under 20 N, after being fixed with double-sided adhesive tape on the lower plate. The evolution of the elastic (storage, G’) and viscous (loss, G”) moduli were investigated via two sets of measurements in the frequency range of 0.01 Hz to 50 Hz for 30 min under a constant temperature of 25 °C.

In order to test the antimicrobial properties of the functionalized textiles, the samples were first sterilized by UV–C irradiation (λ = 245 nm) for 7 min, with an irradiation distance of 2 cm, before being stored in a sterile container. Their antimicrobial activity was quantified via a liquid diffusion assay, while a plate diffusion assay was used to visualize the microbial growth inhibition. The assays were performed in triplicate and for each one, the following samples were tested in duplicate: textiles coated with photoinduced Ag@polymer (3 wt % and 5 wt % silver), textiles coated with photoinduced Ag-free polymer and silicon wafers used as a control material (chosen for its inertness against microorganism proliferation and for the high image quality it provides with upright fluorescence microscopy).

**Liquid diffusion assay.** The samples were placed in individual, sterile dishes and inoculated with 4 mL of *E. coli* or *C. albicans* suspensions of 0.1 OD_600_. Samples in microbial suspensions were placed at 30 °C for *E. coli* and 37 °C for *C. albicans* and incubated for 1 h, 3 h, 6 h, or 24 h. After incubation, microbial growth was evaluated by measuring the suspension’s OD_600_, collected for each material sample. The growth inhibition rate, provided by the release of antimicrobial substances, was then calculated for each material and control sample using Equation 1 and Equation 2. Significance of differences was assessed by the Student’s t-test.


[1] $$\begin{array}{l} {\text{growth inhibition rate}}_{/\text{control material}}\text{ (\%) =}\\ \frac{{\text{OD}}_{{\text{600}}_{\text{control material}}}-{\text{ OD}}_{600_{\text{sample}}}}{{\text{OD}}_{600_{\text{control material}}}}\times 100 \end{array}$$

where 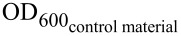 and 
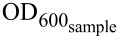
 are the absorbance values of the suspensions collected from the control material and the tested sample, respectively.


[2] $$\begin{array}{l} {\text{growth inhibition rate}}_{/\text{control culture}}\text{ (\%) =}\\ \frac{{\text{OD}}_{{\text{600}}_{\text{control culture}}}-{\text{ OD}}_{600_{\text{sample}}}}{{\text{OD}}_{600_{\text{control culture}}}}\times 100 \end{array}$$

where 
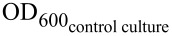
 and 
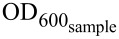
 are the absorbance values of the suspensions collected from the control material and the tested sample, respectively.

**Plate diffusion assay.** Each textile material sample was fixed to a 12 mm diameter glass cover slide using a biocompatible glue (previously tested; data not shown) to keep it flat and subsequently sterilized as described above. Then, 100 mL of fresh *E. coli* and *C. albicans* pre-cultures, prepared as described above, were carefully spread on M63G agar plates. The sterilized material samples were then placed on the resulting microbial film and incubated for 24 h at 30 °C for *E. coli* and 37 °C for *C. albicans*. Bacterial growth around the samples was examined to identify the potential presence of a growth inhibition zone. Pictures of each agar plate were thus taken and analyzed using the FIJI^®^ software in order to reliably measure the area of each sample and the corresponding growth inhibition zone. Results are expressed as mean inhibition area ± standard deviation (SD). The mean area of the samples was 112 ± 7 mm^2^.
